# Recent Advances in One-Dimensional Micro/Nanomotors: Fabrication, Propulsion and Application

**DOI:** 10.1007/s40820-022-00988-1

**Published:** 2022-12-29

**Authors:** Yuhong Zheng, He Zhao, Yuepeng Cai, Beatriz Jurado-Sánchez, Renfeng Dong

**Affiliations:** 1 School of Chemistry, South China Normal University, Guangzhou, 510006 People’s Republic of China; 2 Department of Analytical Chemistry, Physical Chemistry, and Chemical Engineering, Universidad de Alcala, 28871 Alcalá de Henares, Madrid Spain; 3 Chemical Research Institute “Andrés M. del Río”, University of Alcala, 28871 Alcalá de Henares, Madrid Spain

**Keywords:** 1D micro/nanomotors, Fabrication methods, Driving mechanisms, Applications

## Abstract

Due to their tiny size, autonomous motion and functionalize modifications, micro/nanomotors have shown great potential for environmental remediation, biomedicine and micro/nano-engineering. One-dimensional (1D) micro/nanomotors combine the characteristics of anisotropy and large aspect ratio of 1D materials with the advantages of functionalization and autonomous motion of micro/nanomotors for revolutionary applications. In this review, we discuss current research progress on 1D micro/nanomotors, including the fabrication methods, driving mechanisms, and recent advances in environmental remediation and biomedical applications, as well as discuss current challenges and possible solutions. With continuous attention and innovation, the advancement of 1D micro/nanomotors will pave the way for the continued development of the micro/nanomotor field.

## Highlights

- In-depth overview of the classification of one-dimensional (1D) micro/nanomotors and strategies for fabricating them are reviewed.
- Driving mechanisms and progress of 1D micro/nanomotors for applications are summarized.
- Challenges and future prospects of 1D micro/nanomotors are discussed.

## Graphical abstract

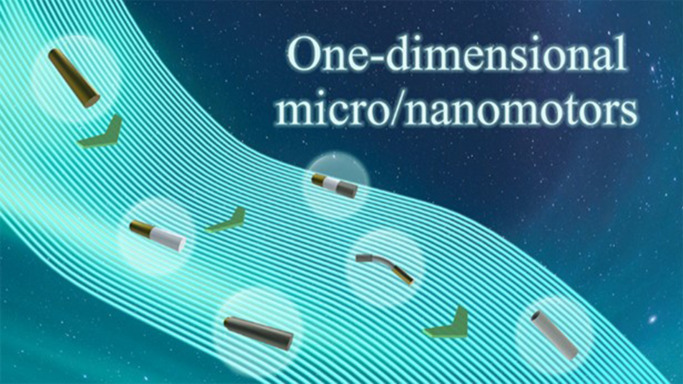

## Introduction

Micro/nanomotors are artificial micro/nanoscale devices that can convert other forms of energy in the environment into mechanical energy for autonomous motion [1–3]. Since the advent of the first gold/platinum (Au/Pt) bimetallic nanorods in 2004 [4], artificial micro/nanomotors have attracted extensive attention of scientists in the field of nanotechnology research. These Au/Pt bimetallic nanomotors could achieve axial motion in a hydrogen peroxide environment, suggesting that micro/nanomotors can convert chemical energy into mechanical energy and thus achieve motion under microscopic conditions. Indeed, the unique features of micromotors such as small size, autonomous movement and functional modification motivated a research upsurge into many disciplines such as chemistry, biology, physics, and medicine, and has become a hot spot in the field of nanoscience research today [5–9].

Among the types of micro/nanomotors, 1D micro/nanomotors are one of the most distinctive [10]. 1D micro/nanomotor is prepared based on 1D micro/nanomaterial. It combines the advantages of autonomous movement and functionalization based on 1D micro/nanomaterial. In general, 1D micro/nanomaterials refer to linear materials with two-dimensional limits below 100 μm or 100 nm, including micro/nanorods, wires, and tubes [11, 12]. Due to its structural anisotropy, 1D micro/nanomaterials not only have excellent performance in optical and electromagnetic sensing but also show great potential in catalytic modification, environmental remediation, nanomanufacturing, and biomedical applications [12–15].The large specific surface area provides an excellent site for functional modification, and the anisotropy of the 1D structure allows the design of 1D micro/nanomotors for different motion modes, such as axial and rotational motion [16]. In addition, the high aspect ratio has facilitated the design of complex structures to broaden their applicability into other fields. Critical variables in the design of 1D micromotors are the use active materials and the synthesis of asymmetric structures. Active materials are usually chemically active, magnetically responsive, electrically responsive, optically responsive, or acoustically responsive materials, etc. [17]. Their function is to convert the corresponding external energy into the force around the motor, while the asymmetric structure serves to break the balance of the force around the motor, which propels the motor directionally and further converts this specific external energy into the mechanical energy of the motor [18, 19]. Therefore, both are indispensable for building 1D micro/nanomotors.

**Fig. 1:**
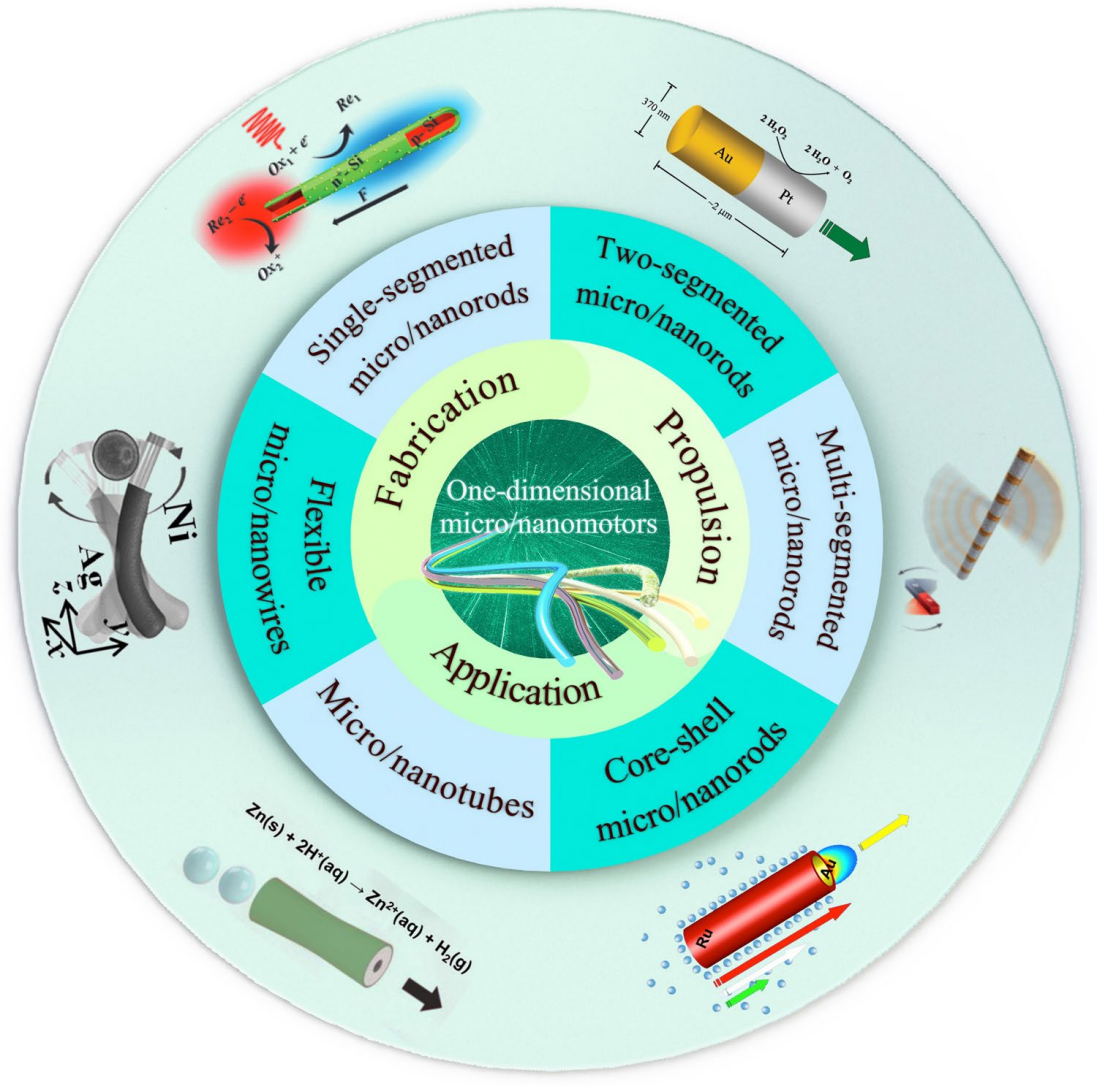
Schematic diagram of common structures, drive principles and applications of 1D micro/nanomotors. Single-segmented micro/nanorods: reprinted with permission from Ref. [20]. Copyright (2014) American Chemical Society. Two-segmented micro/nanorods: reprinted with permission from Ref. [4]. Copyright (2004) American Chemical Society. Multi-segmented micro/nanorods: reproduced with permission from Ref. [21]. Copyright (2017) John Wiley & Sons. Core–shell micro/nanorods: reprinted with permission from Ref. [22]. Copyright (2016) American Chemical Society. Micro/nanotubes: reproduced with permission from Ref. [23]. Copyright (2017) Royal Society of Chemical. Flexible micro/nanowires: reproduced with permission from Ref. [24]. Copyright (2012) John Wiley & Sons

Owing to these unique properties, 1D micro/nanomotors have great potential for applications range from biological applications to environment remediations, such as drug delivery, disease diagnosis and therapy, and pollutant detection or degradation*.* However, micro/nanomotors still have some challenges to be addressed, such as low energy conversion rate, high energy demand, and harmful energy input, which all can greatly limit their operability and application scenarios. In this review we systematically introduce recent advances in 1D micro/nanomotor, including the fabrication methods, typical driving mechanisms and applications in different fields of 1D micro/nanomotors in recent years, and finally summarizes the current challenges and future development trends (Fig. 1).

## Fabrication of 1D Micro/Nanomotors

**Fig. 2:**
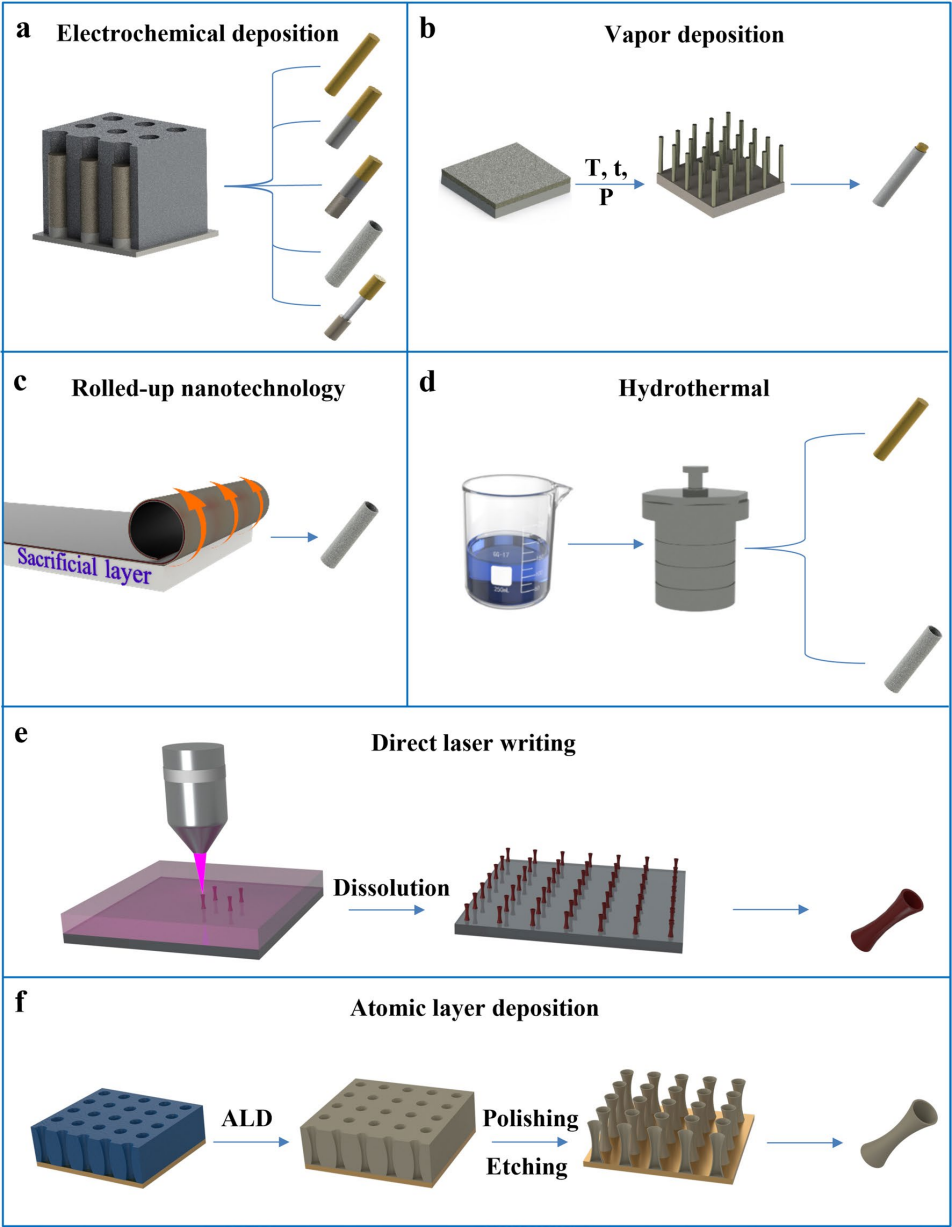
Methods commonly used to prepare 1D micro/nanomotors. **a** Electrochemical deposition technique. **b** Vapor-phase deposition technique **c** Rolled-up nanotechnology. **d** Hydrothermal synthesis technique. **e** Direct laser writing. **f** Atomic layer deposition

In recent years, there are more and more techniques to prepare 1D materials, and researchers have found some suitable methods to prepare 1D micro/nanomotors, including electrochemical deposition techniques, vapor deposition techniques, rolled-up nanotechnology, hydrothermal synthesis techniques, direct laser writing and atomic layer deposition. As shown in Fig. 2, different methods have been adopted for the synthesis of 1D micro/nanostructures of various materials and different morphologies. In the following sections, we summarize several methods to fabricate 1D micro/nanomotors.

### Electrochemical Deposition

Electrochemical deposition refers to a technology, in which a current pass through the migration of positive and negative ions in an electrolyte solution under the action of an external electric field, and a redox reaction of gaining and losing electrons occurs on the electrode to form a coating. Currently, many 1D micro/nanomotors are fabricated by electrochemical deposition techniques, i.e., monometallic nanorods, Janus bimetallic nanorods, tubular micro/nanostructures, and flexible nanowires. Electrochemical deposition allows the synthesis of micro/nanostructures with specific morphologies (such as nanorods, nanotubes, nanospheres, nanosheets) [25, 26]. The electrodeposition fabrication process can be divided into three groupings: no template [27, 28], soft template [29, 30] and hard template [31].

Hard template-assisted electrodeposition is one of the most efficient and convenient methods for the production of nanorod arrays, and the templates used are generally home-made or commercial track-etched films such as anodic aluminum oxide (AAO) and polycarbonate (PC) [32]. When preparing samples, whether to choose an AAO film or a PC film is usually judged by the following three points: (1) The nature of the electrolyte. Since AAO film is not resistant to acid and alkali, and electrodeposition is carried out under the condition of high acidity and alkalinity, PC films must be selected. (2) The distance between the apertures. PC films usually have large pore spacing. (3) The geometry of the pores. AAO films usually form bifurcations at the end of the pore channel, while PC films have cylindrical pores that are hardly inclined, so when using AAO films, it is often necessary to pre-deposit a sacrificial layer to fill up the bifurcations, while PC films do not have this issue. Past the above differences, AAO membrane and PC membrane are both thin films with many pores, and the operations are very similar when used. A typical electrodeposition fabrication process for a 1D micro/nanomotor goes as follows: (1) A layer of conductive metal, usually gold or silver, is deposited on one side of the template by atomic sputtering or other forms to make it conductive [31, 33, 34]. (2) Electrochemical deposition is performed using a three-electrode system or a two-electrode system. (3) The conductive metal layer at the bottom of the template is removed. (4) The template is dissolved to release the prepared 1D material. The double-electrode system consists of a working electrode and counter electrode, while the three-electrode system consists of a working electrode, a counter electrode, and a reference electrode. The working electrode is used to monitor the electrochemical reaction in solution, in other words, is where the electrochemical reaction took place, or the electrons impact [35]. The counter electrode is also called the auxiliary electrode. The auxiliary electrode and the working electrode form a circuit to make the current on the working electrode unblocked, so as to ensure that the reaction studied takes place on the working electrode. When oxidation or reduction reaction occurs on the working electrode, the opposite electrode can be arranged for gas precipitation reaction, or the reverse reaction of the working electrode reaction, so that the composition of the electrolyte remains unchanged. However, the potential of the electrode will change with the change of the current, and polarization will occur when the current is large, which is easy to cause errors. Therefore, a reference electrode with constant potential and not affected by the change of electrolyte composition is introduced to precisely control the potential of the working electrode [36]. In the system of electrodeposited 1D motor, the conductive template after sputtering metal is used as the working electrode, the platinum electrode is generally used as the counter electrode, and the Ag/AgCl electrode is generally used as the reference electrode [32, 36–38]. For example, when Au nanorods are deposited through the above system, AuCl_4_^−^ in the electroplating solution undergoes a reduction reaction on the surface of the working electrode to generate Au [39]. In addition to depositing metal materials, electrochemical deposition can also prepare other materials. For example, when preparing tubular motor by electrodeposition, a polymer layer is usually pre deposited by electro polymerization as a support layer and a protective layer, such as PEDOT [40] and PANI [41]. Researchers are usually select templates, electroplating solutions and reaction time according to the size and type of materials required, so as to control the diameter, composition and length of the final product. Alternative use of different electroplating solutions can obtain 1D motors with different components or structures (Fig. 2a) [42]. For example, single-component gold nanorods [39] can be deposited with gold plating solution, Au/Pt bimetallic nanorods [4] can be prepared by adding platinum plating solution after gold deposition, and the alternating use of gold plating solution and nickel plating solution (Au/Ni)_8_ multicomponent motors [21] can be prepared in repeating plating steps. It is also possible to prepare flexible nanowires [43] or tubular structures [41] by adjusting a suitable parameter to cooperate with the corresponding plating solution and template species.

Overall, the 1D micro/nanomotors fabricated by this template-assisted electrochemical deposition method can have tailored composition, length, and sequence of the micromotors, and the diameter of the motors can be varied based on hard template selection [44–46]. It has the advantages of low chemical environment requirements and simple operation. At the same time, due to operational errors and cost problems, the uniformity of the samples prepared by this method is not good, and it is difficult to prepare motors in large quantities.

### Vapor Deposition

Vapor deposition technology is a new technology that uses physical and chemical processes in the gas phase to change the composition of the substrate surface and form a metal or compound coating with special properties or structures on the surface (Fig. 2b). Generally, the vapor deposition growth of 1D materials can be divided into two categories according to its principle: chemical vapor deposition (CVD) and physical vapor deposition (PVD) [47–49].

CVD is a process that uses gaseous or vapor substances to react on the gas phase or gas–solid interface to generate solid deposits. Among various mechanisms of CVD, the vapor–liquid–solid (VLS) growth mechanism is often used to prepare 1D micro/nanomotors [50]. The VLS process consists of four steps: (1) delivery of gas-phase precursors, (2) adsorption and desorption of precursors on the catalyst surface, (3) diffusion of materials on liquid alloy catalysts, (4) formation of solids in nanorod morphology by precipitation of crystals [51]. For example, Si nanomotors are prepared using the mechanism of VLS. The researchers used SiO_2_ as the substrate, composed and etched with piranha solution to form an array, and then sputtered Pt in the holes of the array as a catalyst. In the high-temperature system of the reaction, the meteorological precursor SiCl_4_ reacts with H_2_ and decomposes into Si atoms which are adsorbed by Pt to form eutectic droplets, and the Si atoms continue to melt into Pt/Si droplets and precipitate due to supersaturation, forming nanomotors based on Si nanorods [52].

PVD technology refers to the technology that evaporates the surface of a material source (solid or liquid) into gaseous atoms or molecules, or partially ionizes into ions, under vacuum conditions through physical methods, and deposits materials with special functions on the substrate surface through a low-pressure gas (or plasma) process [53, 54]. The deposition types include vacuum evaporation [55], sputtering deposition [56], ion plating [57], etc. The vacuum evaporation deposition method is generally used to prepare 1D micromotors. The process includes the following steps: (1) The source material is converted from the condensed phase to the gas phase due to the high temperature. (2) The vapor phase particles migrate from the evaporation source to the substrate through the carrier gas. (3) When the gas particles reach the substrate, they condense, nucleate, and grow into nanorods [58]. For example, Sb_2_Se_3_/ZnO core–shell nanomotors are prepared base on Sb_2_Se_3_ nanorods, which are grown on silicon wafer using Sb_2_Se_3_ powder as evaporation source and high-purity Ar as carrier gas [59]. By selecting a mixed evaporation source, nanorods with different compositions can be obtained at different positions of the substrate. For example, using the mixture of ZnSe and CdSe powders as the evaporation source, Zn_X_Cd_1-X_Se alloy nanorods in Zn_X_Cd_1−X_Se/Cu_2_Se core–shell nanomotors with different stoichiometry can be obtained at different locations, from *x* = 0 (CdSe) to 1 (ZnSe) [60].

Vapor deposition technology can control the composition of products through the source and location of materials. It has the advantage of not discharging waste water, waste gas, waste residue and other pollutants [51]. The preparation process is relatively simple, but the disadvantage is that the instruments employed in the experiment are relatively expensive, and high-purity raw materials are required.

### Rolled-Up Nanotechnology

Rolled-up nanotechnology is a unique method of self-assembly of nanofilms into 3D structures using strain engineering and has been widely used to prepare tubular and helical structures (Fig. 2c) [61–63]. This technique generally achieves the curling of the nanofilm by introducing and manipulating the strain gradient perpendicular to the nanofilm or the external force acting on the film, which generally includes at least the following two steps: (1) Sequentially depositing the required materials on the substrate, such as sacrificial layers and thin-film layers of various materials. (2) Treating the sacrificial layer by different means or apply external force to separate the film layer from the substrate and cause it to curl [64–66].

Due to the different materials and processing methods used, the mechanism of film curling is different. For example, without a sacrificial layer, the film layer and the substrate can be separated by an external force (such as ultrasonic treatment [67] and shaking [66]). The separated film layer is bent due to the residual stress between different material layers, and finally forms a multi-layer tubular structure [67]. With the sacrificial layer, different lattice constants of the materials can be used to stretch and curl other layers by removing the sacrificial layer with smaller lattice constants. It is also possible to reverse the relaxation of the upper and lower layers by controlling the strain gradient to cause a curling effect [68–70]. Using these principles, different material combinations of tubular motors can be fabricated by crimping techniques [71].

Rolled-up nanotechnology has become a versatile method for fabricating size-controlled tubular micromotors [69, 72]. The researchers can control the length and thickness of the tubular motor by controlling the shape of the sacrificial layer and can also control the material type and composition order of the tubular motor by adjusting the number of thin film layers, the material type, and the order of each layer [66, 73]. This technique can batch manufacture tubular micromotors, which has great advantages in material composition and structural design. However, such preparation methods are often expensive and demanding in terms of operation and environment. Meanwhile, due to technical limitations, it is currently difficult to fabricate nanoscale tubular motors by this method.

### Hydrothermal Synthesis

Hydrothermal synthesis is a synthesis method using chemical reactions of substances in aqueous solutions. Generally, in a special reactor (reaction kettle), an aqueous solution is used as the reaction system, and the reaction system is brought to a relatively high temperature and high-pressure state by heating and pressurizing (Fig. 2d) [74, 75]. In this state, substances that are difficult to dissolve under normal conditions are gradually dissolved and have high reactivity that is not present in a normal state, and finally undergo inorganic synthesis [76].

Hydrothermal synthesis generally includes the following steps: (1) Configure an appropriate amount of mixed solution according to the reaction system. (2) Transfer the mixed solution to the reaction kettle and heat it for the corresponding time and temperature as needed. (3) Wash the sample after the reaction system is cooled to room temperature. The hydrothermal growth of different nanostructures mainly depends on the reaction temperature and pressure, the precursors used, and the pH of the solution. Using this method, various 1D motors or motor precursor materials can be prepared, such as TiO_2_ nanorods [77], Ag nanorods [78], and self-assembled microrods composed of melamine and cyanuric acid [79].

Hydrothermal synthesis has considerable potential for growing crystals from solution for various materials and morphologies due to its simple operating steps and low instrumental cost. In addition, hydrothermal synthesis is a one-pot reaction and can be prepared in large quantities. However, compared with several other 1D motor preparation methods, the products prepared by hydrothermal synthesis have poor size uniformity and lack flexibility in material combination and structural design.

### Direct Laser Writing

Direct laser writing (DLW) has been widely used to fabricate structures with micro/nano machining accuracy [80]. In DLW technology, a photoresist is usually deposited on a substrate that can move along a pre-programmed path to expose the photoresist part to the focus of the laser. DLW is mainly divided into two mechanisms: photoablation and photopolymerization. Photoablation uses a positive resist in the laser focus area, which is easily dissolved in a photosensitive developer to retain the unexposed part. On the contrary, photopolymerization refers to the polymerization of negative resists in the laser focus area, which causes them to remain undissolved in the developer solution and retain the exposed part. [81].

As a micro-level fine machining and manufacturing method, DLW technology has been widely used to prepare micromotors. Photoablation has been exploited to fabricate patterned micromotors, while photopolymerization is mainly used to prepare micromotors with complex structures (Fig. 2e). Among them, DLW has been used to prepare 1D micromotors [82]. For example, photopolymer-based tubular micromotors can be manufactured using two-photon polymerization DLW technology [83]. To this end, a negative resist is placed on the glass substrate and cross-linked by a 780 nm laser, and then the tubular structure is constructed through a preset focusing route. Finally, the Pt layer was deposited into the tubular structure by electron beam evaporation, and a chemically driven tubular micromotor was obtained [84].

### Atomic Layer Deposition

Atomic layer deposition (ALD) is a technology that allows to deposit materials layer by layer on the substrate surface as a single atomic film (Fig. 2f) [81]. The chemical precursors are introduced into the substrate surfaces, generating a sub monolayer of the film on an atomic scale. ALD generates a chemical reaction by alternately injecting two or more precursors into the chamber, in which materials are deposited layer by layer on the surface of the substrate under specific temperatures and pressure. Unlike CVD, precursors are not pumped at the same time during ALD but are pumped in sequential pulses [85].

ALD is widely used to manufacture tubular micro/nanomotors due to its outstanding advantages of thickness control, uniformity, and low temperature deposition [86, 87]. For example, ZnO/Pt tubular micromotor can be prepared by ALD technology. First, at 120 °C, a pulse sequence consisting of diethyl zinc and H_2_O precursor was deposited for 1200 cycles to obtain tubular ZnO structure. Then, Pt layer inside tubular structure was deposited by the O_2_-based ALD method at 100 °C using (1, 2, 5, 6-*η*)-1,5-hexadiene dimethyl platinum(II) precursor. The resulting tubular micromotor is propelled by ultraviolet light irradiation in H_2_O_2_ fuel solutions [23].

### Others

Natural structures with unique morphological features such as of pollen or microalgae have been exploited as templates for micromotors [88]. The nanomaterials or catalytic metal responsible for propulsion can be easily incorporated by several approaches including adsorption [89], evaporation [88], chemical plating [90], etc. Natural kapok fiber is a renewable plant with an asymmetric hollow tubular structure and is a natural template for preparing tubular micromotors in large quantities [91].

LbL template assembly is a direct method to fabricate microtubular structures, which is widely used in the preparation of multi-layer tubular structures [92]. Unlike template-assisted electrochemical deposition, LbL-assembled tubes are usually formed by self-assembly triggered by electrostatic force [93], hydrogen bond [94], covalent bond [95], etc. This method is suitable for a variety of materials, especially biocompatible materials, and is also a method for preparing polymer-based multilayer tubular micromotors. By electrostatic effect, positively charged chitosan (CHI) and negatively charged sodium alginate (ALG) are alternately absorbed into the PC membrane with a thickness of about 10 μm and a pore diameter of about 600 nm. After the self-assembly of 18 bilayers, Pt nanoparticles were loaded onto the inner wall of the tube, and then the PC film was dissolved to obtain a polymer-based multilayer tubular nanomotor [92].

Interfacial synthesis is an effective method to control the reaction space [96, 97]. MOF-based nanorods and nanotubes have been synthetized by placing specific templates at the interface of two solvents [98]. On this basis, researchers first introduced magnetic materials before the formation of the 1D structure, and then reduced them to Pt nanoparticles at the end after the formation of the 1D structure, thus building a MOF-based motor with the magnetic response and special functions [99].

Etching is a technique that removes unwanted material parts by physical or chemical means [100]. Etching technology includes chemical etching [101, 102], plasma etching [103, 104], laser etching [105, 106] and other etching technologies [107–109]. Among them, chemical etching and laser etching have been applied to prepare 1D motors. For example, Si nanomotors can be obtained by etching patterned silicon wafers with HF and H_2_O_2_ [110].

## Propulsion Mechanism of 1D Micro/Nanomotors

Precise control of the motion of micro/nanomotors is critical for their future applications. In the absence of external stimuli, micro/nanomotors typically undergo weak Brownian motion. In the presence of external stimuli, such as magnetic fields, ultrasonic fields, electric fields, light or chemical changes, the motion of micro/nanomotors can be controlled, directed, and even toggled. The driving of the 1D micro/nanomotor varies with the material and structure of the motor itself. Due to the particularity of its materials and structures, 1D micro/nanomotors can be designed into motors with different driving mechanisms according to the combination of materials. In the next section, we will summarize the respective driving methods of 1D micro/nanomotors with different structures.

From the early stage of micro/nanomotor research to the present, researchers have developed various 1D micro/nanomotors, ranging from simple single segment nanorods, double segment nanorods to microtubes, core–shell nanorods and multi segment flexible nanowires, etc. Next, we describe the 1D micro/nanomotor recently reported by researchers based on materials, structures and driving methods. The summary is shown in Table 1 at the end of this review.

**Table 1:** 1D micro/nanomotors with different shapes and materials

Shape		Material	Propulsion mechanism	References
Single-segmented micro/nanorods		Au	Acoustically driven	[111]
		Cu	Electrically driven	[112]
Two-segmented micro/nanorods		Au/Ru	Acoustically driven	[113]
		Co/Pt	Chemically driven	[114]
		Au/TiO_2_	Light driven	[115]
		Pt/Ag	Light driven	[116]
		Si/TiO_2_	Light driven	[117]
		JFRs	Chemically driven	[118]
		Pt/SiO_2_	Chemically driven	[119]
		Au/Pt	Chemically driven	[120]
		Nanoclay/Pt	Chemically driven	[121]
		Si/Pt, Si/Ag	Chemically driven	[122]
		Au/ZnO	Light driven	[123]
		Au/Fe_2_O_3_	Light driven	[124]
		Au/ Ni	Acoustically/ Magnetically driven	[125]
Multi-segmented micro/nanorods		Ru/Ni/Au	Acoustically driven	[126]
		Au/Fe/Ni	Chemically driven	[127]
		CdSe/Au/CdSe	Electrically driven	[128]
		(Au/Ni)_8_	Magnetically driven	[21]
		Pt/Ni/Au/Ni/Au	Chemically driven	[129]
Core–shell micro/nanorods		Zn_X_Cd_1−X_Se/Cu_2_Se	Light driven	[60]
		Au/Ru	Light driven	[130]
		Au/Ag	Light driven	[131]
		Sb_2_Se_3_/ZnO	Light driven	[59]
Micro/nanotubes		PEDOT/Zn	Chemically driven	[132]
		PANI/Zn	Chemically driven	[41]
		Cu/Ag	Chemically driven	[133]
		Fe/Pt	Chemically driven	[134]
		TiO_2_/Pt	Chemically driven	[135]
		g-C_3_N_4_	Light driven	[79]
		(Pb_0.25_Ba_0.15_Sr_0.6_)TiO_3_	Chemically driven	[136]
Flexible micro/nanowires		Au/Ag/Ni	Magnetically driven	[137]
		Ni/Au/PPy, Ni/hinge/Rh	Magnetically driven	[138]
		Ni/Ag	Magnetically driven	[24]

### Single-Segmented Micro/Nanorods

**Fig. 3:**
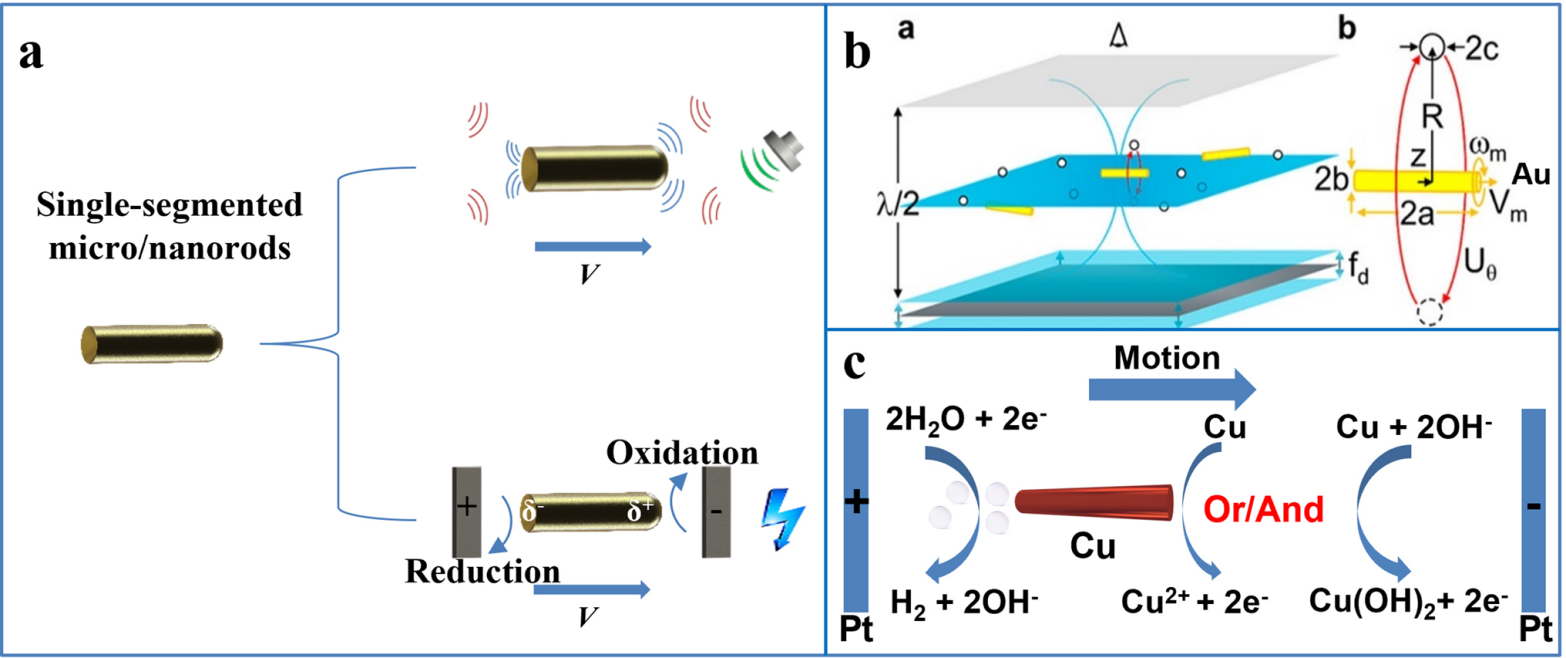
**a** Single-segmented nanorods driven in ultrasonic fields and electric fields. **b** Driving mechanism of the gold nanorod motors in the acoustic field. Reprinted with permission from Ref. [20]. Copyright (2014) American Chemical Society **c** Driving mechanism of the Cu nanorod motors in the electric field

The single-segment 1D micro/nanomotor is a rod-like structure composed of one material. Its structure is simple and the preparation method is relatively simple. Generally, it is prepared by template-assisted electrochemical deposition. Due to the unification of materials and structures, the choice of this motor driving method is also limited: the current reports are mostly acoustic field driving and electric field driving (Fig. 3a).

Ultrasonic energy comes from high-frequency sound waves generated by ultrasonic transducers. Ultrasound energy has good directionality, strong penetrating power and little harmful effect on the human body, so it has been widely used due to its high biocompatibility. High-frequency sound waves, especially in the megahertz range, are powerful tools for particle manipulation. Ultrasonic driving is an ideal driving method for micro/nanomotors. Compared with chemical driving, ultrasonic driving does not need to add fuel, which has great advantages in liquid environment. Another significant advantage compared with magnetic and electric field driven motors is that ultrasonic field driven motors are simple to operate, do not require the introduction of magnetic materials, and have a larger usable range. Ultrasonic-driven nanomotors often require asymmetric geometries to facilitate motion by establishing pressure gradients, microfluidics, or bubble cavitation. Ultrasonic actuation provides a powerful propulsion strategy, fast movement, and pre-concentration capabilities. However, it has difficulties in independently manipulating individual nanomotors and is limited by specialized fabrication equipment and limited chamber size.

The researchers systematically studied the ultrasound-responsive rod-shaped structure prepared by template-assisted electrochemical deposition and found that one end of the rod-shaped structure is a concave surface, while the other end is a convex surface or a flat surface. This uneven structure in the sound field results in an asymmetric distribution of the sound pressure scattered by the incident sound wave from the metal surface, which is suitable for acoustic field driving [139]. Concave and convex surfaces reflect acoustic waves differently, just as concave and convex mirrors reflect light, with acoustic waves reflecting toward a point in the concave surface and diverging around in the convex surface, so that the nanorods obtained by electrodeposition are subject to asymmetric thrust in the plane of the sound field. When nanoparticles are suspended in a liquid and subjected to an ultrasonic field, the particles experience several stable, time-averaged hydrodynamic forces known as acoustic radiation pressure or acoustic radiation force. The acoustic radiation force can be divided into primary radiation force (PRF) and secondary radiation force (SRF). Among them, PRF is mainly due to the force generated by the sound field standing wave, which affects some pressure nodes or antinodes, causing particles to migrate or aggregate on a certain plane (Fig. 3b) [20]; SRF is the force generated by sound waves scattered by nearby particles, weaker than the maximum axial PRF, and can cause particles to attract or repel each other [111, 139–142]. In a chamber of a sound field experiment, the standing wave can be calculated according to the following formula:

1 $$ h = \frac{1}{2}n\lambda = \frac{1}{2}n\frac{c}{f},\;n = 1,2,3 $$

In this formula, *h* is the height of the chamber, *c* is the speed of sound in the medium, *λ* is the wavelength, and *f* is the frequency [139]. At the resonant frequency, a nodal plane with the smallest sound pressure will be formed. Researchers can calculate the resonance frequency of different devices according to this formula and adjust it as needed based on the experiment.

When the 1D micro/nanomotor enters this pressure node, the unbalanced sound pressure due to the asymmetry of the two ends will propel the motor, and its motion speed can be calculated by the following model:

2 $$V=\epsilon \mathrm{Re }{\widehat{V}}^{\perp }{\nu }^{\left(\mathrm{1,1}\right)}$$

Reynolds number ($\mathrm{Re}\ll$ 1), near-sphericity ($\epsilon \ll$ 1), ${\widehat{V}}^{\perp }$ denotes the amplitude of the transverse oscillations and ${\nu }^{\left(\mathrm{1,1}\right)}$ is of order one [143]. Researchers often use the gold nanorods prepared by ultrasonic-driven electrodeposition for cargo transportation [144], detection [145] and other applications [146]. There are multiple advantages to such motors, because the gold surface can easily be functionally modified, the preparation of gold nanowires by electrodeposition is relatively simple, and the motors have high biocompatibility due to their use of ultrasonic driven propulsion [111, 140].

In addition to ultrasonic drive, the single-segmented 1D motor can also move in an electric field. When a conductive object is placed under a strong electric field between two electrodes, a polarization that is proportional to the electric field and the characteristic dimension of the object occurs. The potential difference generated across the object can be inferred from the following equation:

3 $$\Delta {V}_{\mathrm{min}}=Ed$$

where *E* is the total external electric field and *d* is the distance across the object. When the polarization voltage is at a suitable value, redox reactions can occur at both ends of the object, which is due to bipolar electrochemistry on micro-objects [147]. Using this principle, when the copper nanorod motor is placed in the electric field in water, because the oxidation potential of copper is lower than that of water, it is oxidized to Cu^2+^ ions (Fig. 3c). Therefore, there is no bubble at the oxidation end. The reaction of water molecules at the reduction end will release H_2_, and finally form a bubble driven motor [112].

### Two-Segmented Micro/Nanorods

**Fig. 4:**
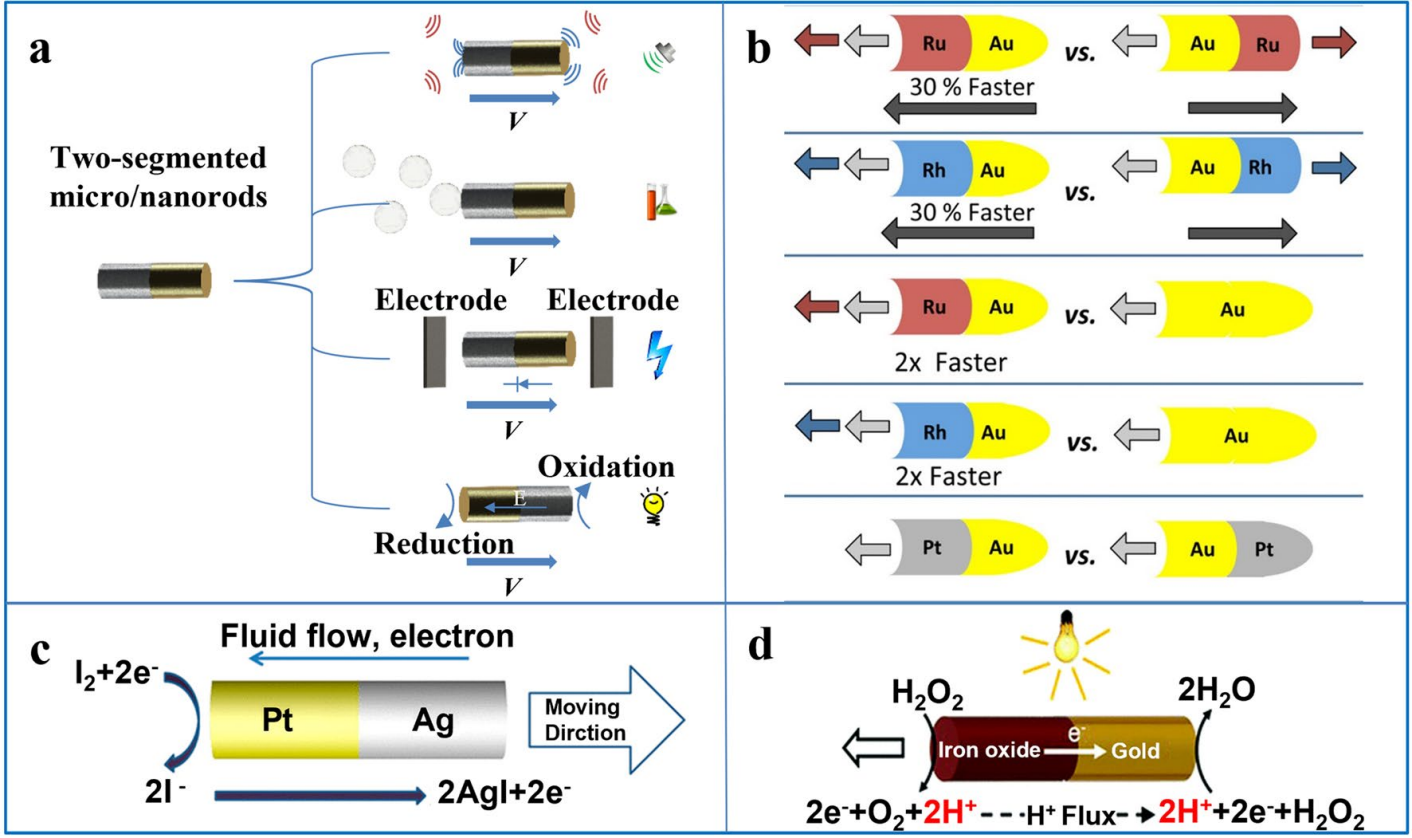
**a** Two-segment nanorods driven in ultrasonic fields, chemical fuels, electric field and light. **b** Motion of Au/Ru Janus nanomotors in the ultrasound field. Reprinted with permission from Ref. [113]. Copyright (2016) American Chemical Society. **c** Pt/Ag Janus nanomotors generated from electrophoretic propulsion motors under illumination while regenerating the fuel. Reprinted with permission from Ref. [116]. Copyright (2016) American Chemical Society. **d** Driving mechanism of Fe_2_O_3_/Au Janus nanomotors in hydrogen peroxide environment. Reproduced with permission from Ref. [124]. Copyright (2017) Royal Society of Chemical

Compared with single-segment micro/nanorods, the two-segment 1D micro/nanomotor is composed of two materials to form a 1D structure with material asymmetry, which gives researchers more maneuverability. Two-segmented nanorods, which are also called Janus structures after the two-faced Roman god, are an emerging nanostructure [148]. This asymmetric structure can achieve some goals that are difficult to achieve with uniform particles. For example, a combination of materials with different functions or properties can be designed so that the motor exhibits directional chemical reactions, photochemical effects, or magnetic responsive behavior. The asymmetry of this material can also be used to combine ultrasonic driving with other driving methods to design motors with multiple driving methods, magnetic conduction motors or motors with other characteristics (Fig. 4a).

As mentioned above, the 1D Janus motor prepared by template-assisted electrochemical deposition has high flexibility in structural design, so it is possible to find a suitable driving method for a particular application according to the composition of the motor material and its functional characteristics. Specific material combinations can also be designed according to experimental needs. In general, 1D Janus motors composed of different materials can have different characteristics and driving principles.

For example, the difference in density between two materials resulting in the order in which they are combined will affect the actuation of motors in the sound field, such as Au/Ru Janus motors. In addition to being able to be driven in the sound field as previously described, the difference in density between the two metals and the order in which the materials are bonded can also affect the motion of the motor (Fig. 4b) [113]. On the other hand, it is also possible to design specific motors by taking advantage of the different chemical properties of the materials. For example, designing a metal catalyst on one end of the Janus structure allows the preparation of chemically driven motors. Chemically driven Janus micro/nanomotors can utilize localized chemicals to obtain driving force from specific catalytic or spontaneous reactions in fluids. This requires the integration of the reactants in the catalytic system into the system, or the conversion of the corresponding substances into fuels. In the design of these fuel-driven micro/nanomotors, a wide variety of substances such as hydrogen peroxide (H_2_O_2_) [149, 150], glucose [151], urea [152] and other chemical fuels [153, 154] have been studied. Among them, H_2_O_2_ is the most widely used fuel, which can be attributed to the fact that many metals can catalytically convert H_2_O_2_ into water and oxygen (O_2_) to form bubbles or concentration gradients that drive the motion of micro/nanomotors [121]. In the chemically driven 1D Janus motor, and even for same-material motors, the driving mechanism can vary substantially from motor to motor. For example, the principle of self-electrophoresis of Pt/Ag Janus nanomotor in I_2_ solution is motion caused by ion surges occurring due to uneven distribution of ionic charges generated by the motor during the reaction process and the formation of micro-electric fields around the motor (Fig. 4c) [116]. The bubble driving mechanism of Co/Pt Janus nanomotors in H_2_O_2_ solution is formed by the asymmetric thrust of metal platinum catalyzing H_2_O_2_ to generate bubbles [114].

Light-driven motors can also be propelled by self-electrophoresis. Light is a renewable energy source, which is an advantage of light-driven micro/nanomotors. All light driven motors are designed based on two factors: material and light source. The essence of light driving is that the motor has asymmetric light response behavior under the light field such that various gradient fields are formed around the motor, and the motor can convert light energy into its own mechanical energy. Various photoactive materials designed as light-driven micro/nanomotors, such as photocatalytic materials, photothermal materials, and photo deformable materials, can produce a series of changes after absorbing light energy, such as chemical reactions or asymmetrical energy fields, which powers the motor. For example, the unique band gap value of semiconductor nanorods makes them photo-responsive materials corresponding to wavelengths, such as ZnO, TiO_2_ and Fe_2_O_3_, which are typical semiconductor materials and are good raw materials for the preparation of light-driven 1D micro/nanomotors. The speed of this light-driven micro/nanomotor can be expressed by the following formula:

4 $$U=b\nabla Y$$

In this formula, *b* is the velocity coefficient, and ∇*Y* is the gradient of the potential function *Y*, such as pressure, temperature, potential, or solute concentration [19]. According to this formula, the greater the gradient around the motor, the greater the speed of the motor, so the design of light-driven micro/nanomotors can be carried out around how to generate various gradients and how to generate larger gradients. These gradient fields can be divided into the gradient field of molecular concentration, the gradient field of ion concentration, and the thermal gradient field according to the driving principle. The thermal gradient field is generally used to drive the micro/nanomotor with a spherical asymmetric structure or tubular structure. The molecular concentration gradient field is due to the uneven distribution of the molecules generated by the photocatalytic reaction around the micro/nanomotor. However, the overall environment always tends to be balanced, so the micro/nanomotor will be driven when the surrounding high-concentration particles diffuse to the low-concentration area [155]. For example, WO_3_/Ag micromotor can generate the concentration gradient of electrolyte O_2_ under light, which causes the motor to be driven by a diffusion swimming mechanism. In addition to generating nonelectrolyte gradients, WO_3_/Ag micromotor can also generate electrical gradients and be driven by a self-electrophoresis mechanism [156]. The gradient field of ion concentration required for self-electrophoresis is due to the uneven distribution of anions and cations generated by the reaction. The asymmetrical distribution of anion and cation will generate a local micro-electric field around the micro/nanomotor. The electronic transition and transfer due to light inside the motor will also form another micro electric field inside the motor, and the interaction with the external micro electric field will drive the micro/nanomotor [157]. For example, the researchers designed an asymmetric ZnO/Au Janus micromotor, which undergoes self-electrophoresis by UV light irradiation in an H_2_O_2_ environment, similar to the self-electrophoresis of TiO_2_/Au micromotors [158]. The driving force of the movement is the self-generated electric field generated by the proton concentration gradient in the H_2_O_2_ photocatalytic reaction [123]. Similarly, the Au/Fe_2_O_3_ nanomotor can decompose hydrogen peroxide under visible light to generate an electric gradient, and then move in the direction (Fig. 4d) [124].

Two-segmented 1D motors can also be designed to be electric field driven. In addition to the driving principle of bipolar electrochemistry, the electric field-driven motor consists of a two-component or even multi-component motor as a diode in an AC electric field, and the entire AC electric field will be locally converted into a DC voltage by the diode, resulting in electrophoretic motion. The speed of motion of such a motor in an AC electric field can be expressed by the following equation:

5 $${U}_{\mathrm{ep}}=\frac{\varepsilon {\varepsilon }_{0}{\zeta }_{\mathrm{p}}}{2\eta }\left({E}_{\mathrm{ext}}-{E}_{d0}\right)$$

where $\varepsilon$ is the dielectric constant of the solvent, $\eta$ is the viscosity of the solvent, ${\zeta }_{\mathrm{p}}$ is the electrokinetic potential (zeta-potential) of the particle, ${\varepsilon }_{0}$ is the dielectric constant of vacuum, ${E}_{d0}$ is the offset field that characterizes the particular pn-junction, and ${E}_{\mathrm{ext}}$ is the applied AC electric field on the electrodes. According to this principle, the Cd/PPy nanorod motor can be propelled forward in a controllable manner under the influence of an AC electric field [128]. This fuel-free, harmful product-free 1D motor enhances biocompatibility for in vitro and in vivo applications.

### Multi-Segmented Micro/Nanorods

**Fig. 5:**
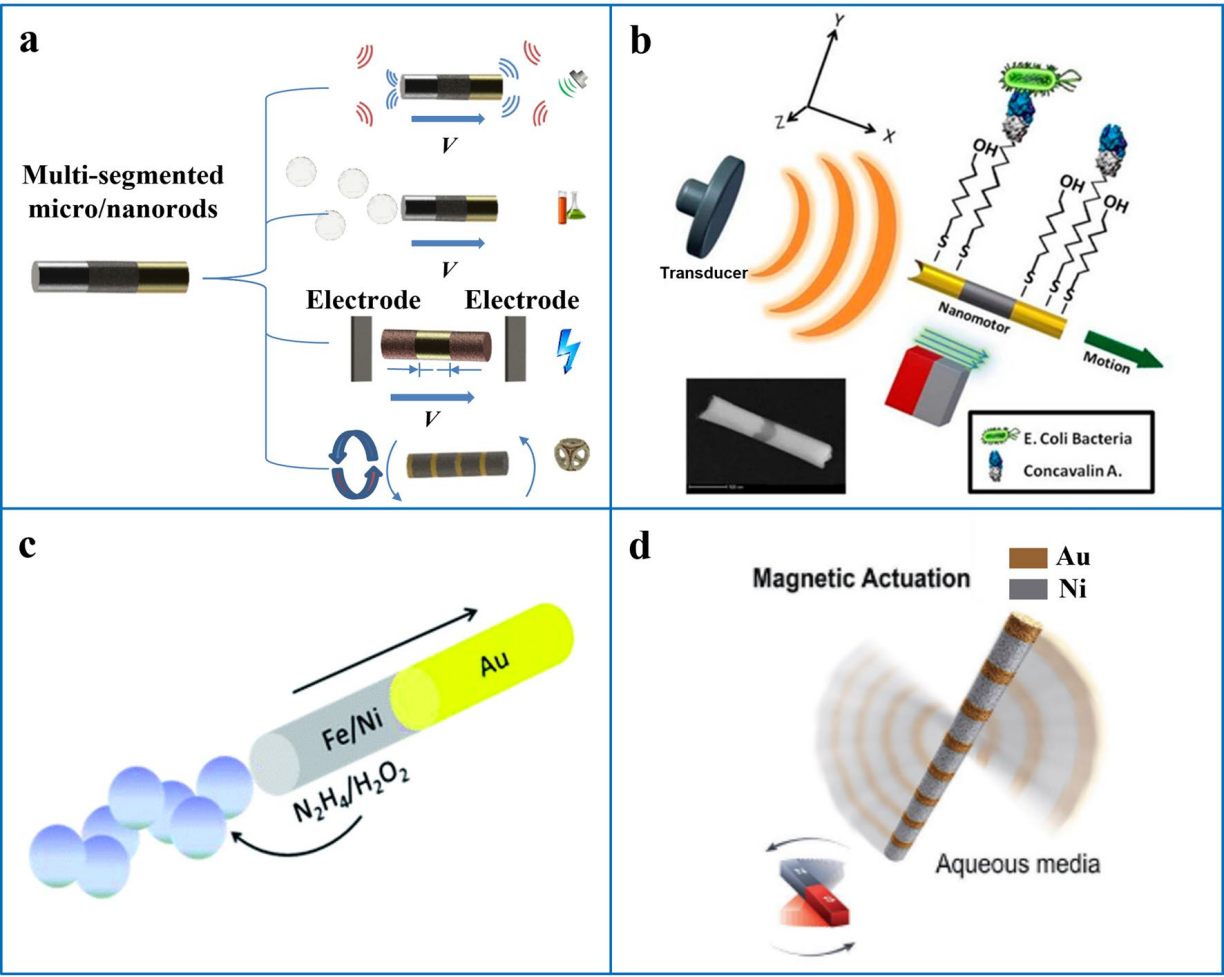
**a** Multi-segment nanorods driven in ultrasonic fields, chemical fuels, electric field and magnetic fields. **b** Motion of Au/Ni/Au nanomotors in the ultrasonic field. Reprinted with permission from Ref. [159]. Copyright (2013) American Chemical Society. **c** Schematic illustration of the driving of the Au/Fe/Ni alloy nanomotor in a mixed fuel composed of hydrogen peroxide and hydrazine. Reproduced with permission from Ref. [127]. Copyright (2011) Royal Society of Chemical. **d** Schematic diagram of the magnetic response of the (Au/Ni) _8_ nanomotor. Reproduced with permission from Ref. [21]. Copyright (2017) John Wiley & Sons

Compared with the Janus two-segment straight nanorod motor, the multi-segment straight nanorod motor is a 1D motor that integrates more materials. It forms surfaces with different functional and physicochemical properties that respond differently to external stimuli (Fig. 5a). In structural design, different functions are generally constructed by choosing different materials. For example, Au/Ni/Au nanorod motors fabricated by electrochemical deposition can be driven in an acoustic field and, due to the integration of metallic Ni, can also navigate in a magnetic field (Fig. 5b) [159]. In a similar structure, the CdSe/Au/CdSe nanorod motor can also act as a diode to realize electrophoretic motion in an AC electric field [128]. The combination of different materials can also produce different effects. For example, an Au/Fe/Ni alloy nanomotor combined with two magnetic materials can be magnetically guided while being driven by gas bubbles in a mixed fuel composed of hydrogen peroxide and hydrazine (Fig. 5c) [127]. The multi-segment (Au/Ni)_8_ nanomotor using a magnetic material uses a combination of different materials to reduce the remanence value of the motor, thereby reducing the magnetostatic interaction and reducing the mutual attraction between the motors (Fig. 5d) [21].

### Core–Shell Micro/Nanorods

**Fig. 6:**
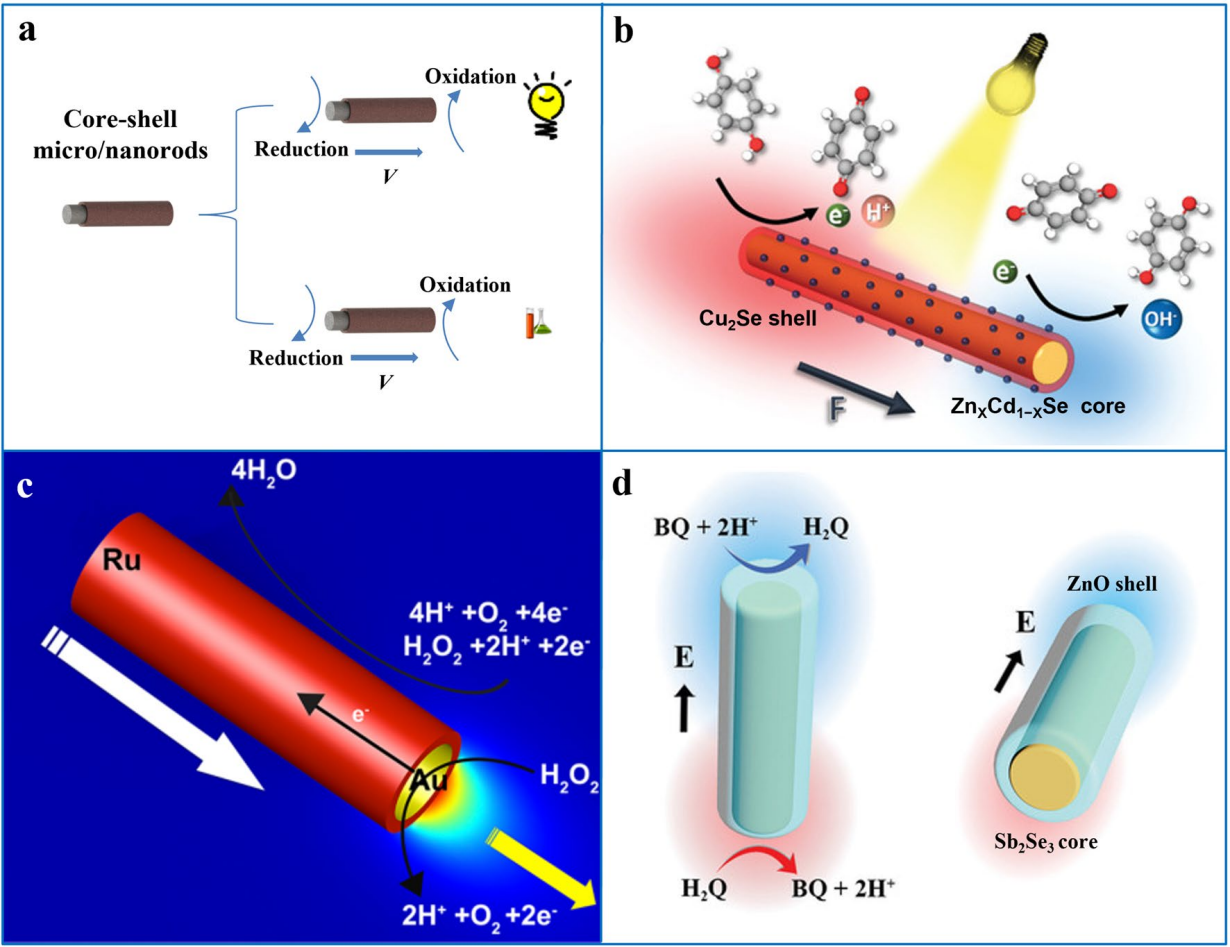
**a** Schematic diagram of a core–shell structured nanorods driven under light. **b** Schematic diagram of photochemically induced electrophoresis-driven Zn_X_Cd_1−X_Se/Cu_2_Se core–shell nanomotor. Reproduced with permission from Ref. [60]. Copyright (2019) Wiley–VCH. **c** Schematic illustration of the driving of the Au/Ru core–shell nanomotor in a hydrogen peroxide environment. Reprinted with permission from Ref. [130]. Copyright (2016) American Chemical Society. **d** Schematic diagram of the photocatalytic decomposition of BQ and driving the motor by the Sb_2_Se_3_/ZnO core–shell nanomotor. Reproduced with permission from Ref. [59]. Copyright (2019) Wiley–VCH

1D core–shell structure is another strategy for integrating different materials besides two-segment and multi-segment structures, which is an ordered assembly structure formed in which a rod-like structure is coated by another material. In terms of structural design, the outer material can play a protective role and prevent the interaction of certain materials with the surrounding environment. Furthermore, like the Janus structure, the heterogeneous interfaces between different components in core–shell micro/nanomotor systems may generate new properties. For example, researchers designed 1D motors with different core–shell structures based on a unique charge migration feature (Fig. 6a). In terms of material selection, the compositions of the core layer and the shell layer has an impact on various aspects such as function and performance. For example, a core–shell nanomotor with an inner Zn_*X*_Cd_1−*X*_Se inner core and an outer Cu_2_Se shell induces charge migration under illumination, which triggers different redox reactions on different surfaces, leading to the self-electrophoresis of the motor (Fig. 6b) [60]. Similarly, Sb_2_Se_3_/ZnO core–shell nanorod motors can also decompose organic substrates after absorbing polarized light and provide a thrust for the migration of nanomotors (Fig. 6d) [59]. In contrast, the three 1D core–shell motors of Au/Rh, Rh/Au, and Au/Ru have different effects on the electroosmotic flow and diffusive electrophoresis in the fuel-driven process due to different material types and structural designs, resulting in differences in the motor behavior of motors of different types and lengths (Fig. 6c) [130].

### Micro/Nanotubes

**Fig. 7:**
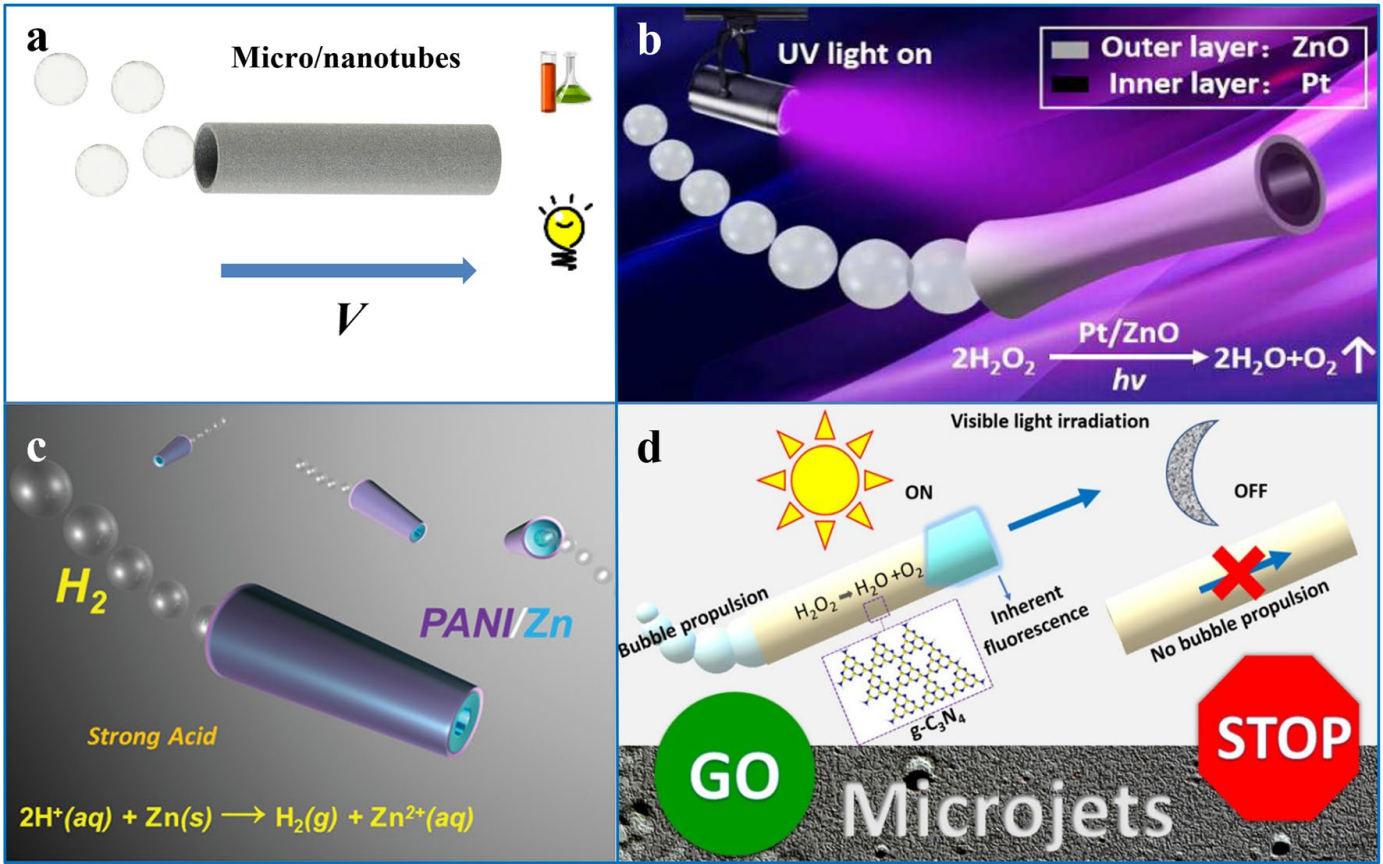
**a** Schematic diagram of bubble driven tubular micro/nanomotors. **b** ZnO/Pt tubular micromotor driven in a chemical environment. Reproduced with permission from Ref. [23]. Copyright (2017) Royal Society of Chemical. **c** PANI/Zn tubular micrometer motor driven in a strong acid environment. Reprinted with permission from Ref. [41]. Copyright (2012) American Chemical Society. **d** Driving of a g-C_3_N_4_ tubular micrometer motor in a light field. Reprinted with permission from Ref. [79]. Copyright (2018) American Chemical Society

Tubular micro/nanomotors have a hollow rod-like structure that is different from the asymmetric materials and structures introduced before, where different materials are integrated radially rather than axially. One of the features of this structure is the large specific surface area, the inner and outer surfaces can be designed with different functions, and the inner space can be used to load goods. Such motors are typically fabricated by roll-up nanotechnology or electrochemical deposition and move in a bubble-driven mode, where bubbles are typically ejected from inside the tubular structure to obtain propulsive force (Fig. 7a).

Tubular motors also have different propulsion mechanisms depending on the composition of the material. The inner and outer surfaces of the tubular motor made of a single material are in contact with the fuel, so the material must either react directly with the fuel to produce gas or be triggered by some stimulus to produce gas. Due to the spatial confinement effect inside the tubular structure, the gas molecules generated in the inner space of the tube are more likely to aggregate to form bubbles, while the open system outside is conducive to the diffusion of gas molecules and is difficult to form bubbles. In this case, when the motor is in a fuel system with a suitable concentration, the bubbles are only formed inside the tube and then extruded from the single opening to propel the motor, such as the TiO_2_ tubular micromotor [22] or the tubular micromotor based entirely on graphite carbon nitride (Fig. 7d) [79]. High or stimulus energies are too high to cause an overly aggressive response, and bubbles may also form around the tubular structure, resulting in erratic movement.

The multi-layer tubular motor can also be divided into two types. The first is a tubular motor in which only the inner layer material participates in the reaction, and the other is a tubular motor in which both the inner layer and the outer layer material participate in the reaction. The outer material of the first tubular motor is usually a very chemically stable material, which shields the inner material from double-sided contact with the fuel, and on the other hand supports the inner material to make the tubular structure more stable. In the tubular motor of this structure, only the inner layer material reacts to generate air bubbles, such as the PEDOT/Zn tubular motor [64] and the PANI/Zn tubular motor (Fig. 7c) [41]. The average velocity of this type of bubble-driven tubular 1D motor can be calculated by the following equation:

6 $${\nu }_{j}^{\mathrm{ave}}=\frac{9nC{R}_{j}L}{3{R}_{\mathrm{b}}^{2}+L{R}_{\mathrm{b}}/\left(\mathrm{ln}\left(\frac{2L}{{R}_{j}}\right)-0.72\right)}$$

where $L$ is the length of the tubular motor, $2\pi Rj$ is its width, $n$ is the rate constant related to bubble generation, and ${\nu }_{j}^{\mathrm{ave}}$ is the average velocity of the tubular micro/nanomotor, $C$ is the concentration of fuel in the solution, and ${R}_{\mathrm{b}}$ is the radius of the bubble [10].

The other is a tubular motor where both the inner and outer layers react. For example, in the ZnO/Pt tubular motor system, ultraviolet light irradiates the ZnO catalytic decomposition of H_2_O_2_ and transfers its electrons to one end of metal Pt. These photogenerated electrons react inside the microtubule to generate H_2_O_2_, which will increase the fuel concentration in the tube and enhance the reaction of Pt catalytic decomposition of H_2_O_2_ (Fig. 7b) [23]. Therefore, the addition of materials such as photocatalysts and quantum dots can facilitate the driving of tubular motors. In addition, tubular micromotor is one of the ideal models driven by photothermal mechanism. The narrow space inside the tubular structure is conducive to limiting the diffusion of heat, resulting in an obvious internal and external thermal gradient to achieve photothermal driven [160].

### Flexible Micro/Nanowires

**Fig. 8:**
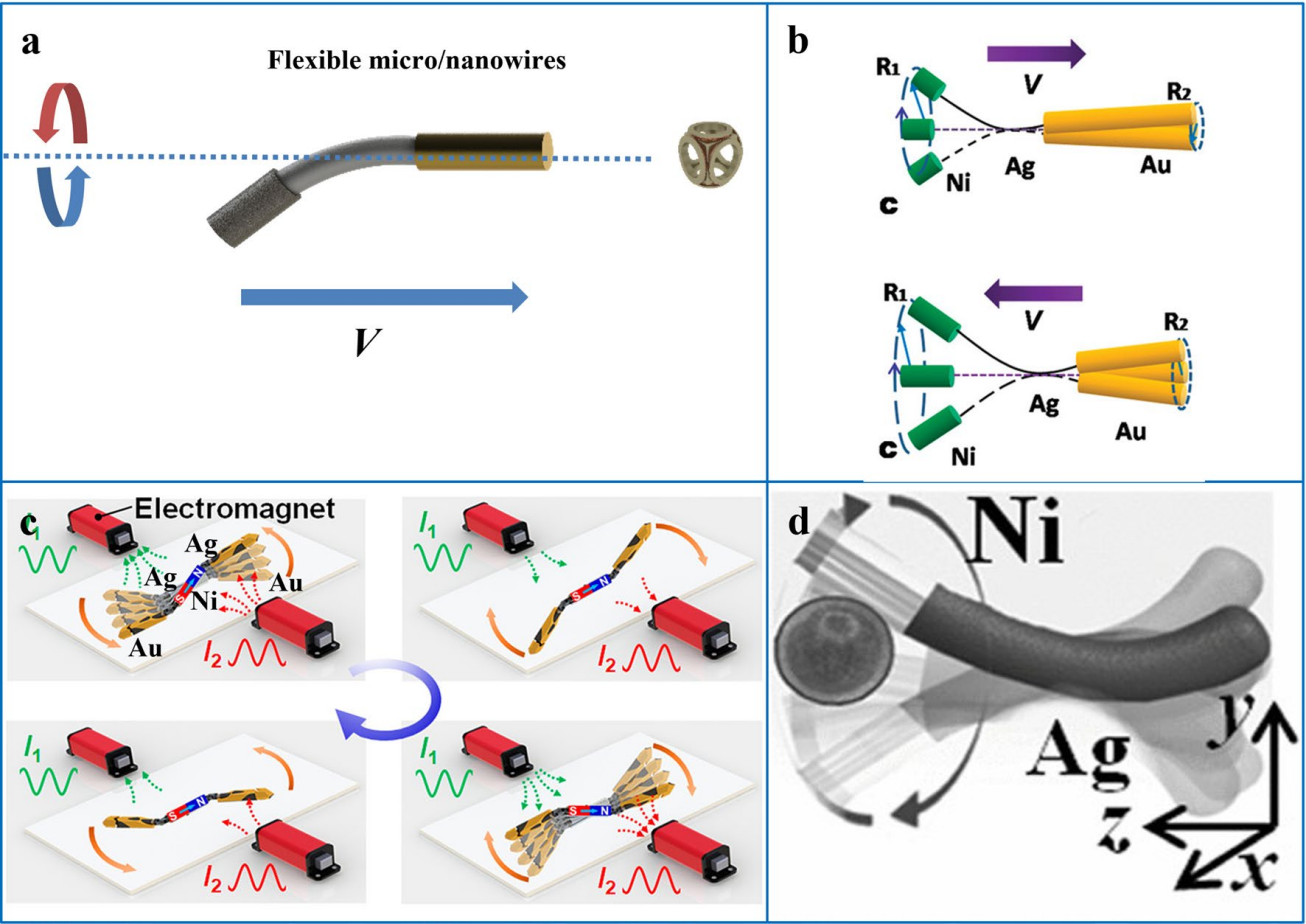
**a** Schematic diagram of a flexible nanowire driven in a magnetic field. **b** Schematic diagram of the motion of the Au/Ag/Ni flexible nanomotor in a rotating magnetic field. Reprinted with permission from Ref. [43]. Copyright (2010) American Chemical Society. **c** Schematic diagram of the motion of the Au/Ag/Ni/Ag/Au flexible nanomotor in an oscillating magnetic field generated by a pair of electromagnets. Reprinted with permission from Ref. [137]. Copyright (2021) American Chemical Society. **d** Schematic illustration of the Ni/Ag flexible nanomotor moving and drug loading in a rotating magnetic field. Reproduced with permission from Ref. [162]. Copyright (2012) John Wiley & Sons

Unlike the rigid structures introduced earlier, there are also flexible 1D micro/nanomotors. Flexible micro/nanomotors are nanowires composed of rigid 1D structures and flexible 1D structures, usually driven by magnetic fields. Magnetic field-driven micro/nanomotors generally require the design of magnetic materials (ferromagnetic, ferrimagnetic, or superparamagnetic materials) as part of the motor, which only requires relatively low magnetic fields to drive. The use of magnetic fields to drive motors first showed excellent biocompatibility under various conditions, while magnetic fields can be controlled in different ways (rotation, oscillation, cone, and gradient fields) to control motors to exhibit different motion mechanisms. The flexible motor is generally driven by a rotating magnetic plane or an oscillating magnetic field, and its structure is generally composed of two or more materials, and the 1D structure in which the multiple materials are combined in a certain order is usually prepared by electrochemical deposition. Among them, magnetic materials and flexible materials are indispensable, and some researchers will also design some non-magnetic rigid materials. When a magnetic object is exposed to a uniform magnetic field, its easy axis of magnetization will automatically align with the direction of the magnetic field. Then in a rotating magnetic field or an oscillating magnetic field whose direction of the magnetic field changes constantly, the easy magnetization axis of the magnetic object will also change its direction, in other words, the magnetic object will change its orientation continuously in the changing magnetic field. When the magnetic motor moves in the changing magnetic field, the rotational force or torque (${\overrightarrow{\tau }}_{\mathrm{M}}$) it receives can be calculated using the following formula:

7 $${\overrightarrow{\tau }}_{\mathrm{M}}={\mu }_{0}\nu \overrightarrow{M}\times \overrightarrow{H}=\nu \overrightarrow{M}\times \overrightarrow{B}$$

where ${\mu }_{0}$ (T · m · A^−1^) is the permeability of free space, $\nu$ (m^3^) is the magnetic volume of the object, $\overrightarrow{M}$ (A · m^−1^) is the magnetization of the object, $\overrightarrow{H}$ (A · m^−1^) is the strength of the external rotating magnetic field, and $\overrightarrow{B}$ (T) is the magnetic induction [161]. The easy axis of a magnetic object is usually defined by the magnetic shape anisotropy, in other words the shape determines the easy axis. A rotating or oscillating magnetic field can cause a magnetic object to translate, if the shape and structure are properly designed. A precisely designed combination of the rigid structure and the flexible structure of the flexible 1D motor is well-suited for variable-direction magnetic field drive. The motor of this structure drives the flexible segment to deform by the response of its magnetic segment in a magnetic field. Due to the different forces at both ends of the flexible deformation, the movement amplitudes at both ends of the deformation will also be different, so as to realize the driven motion (Fig. 8a).

Flexible motors exhibit different deformations depending on the structural design and the type of magnetic field. In the oscillating magnetic field, the magnetic material oscillates to drive the flexible material to deform, and the flexible material will also change the originally symmetrical swing into an asymmetrical swing, and finally realize the overall swing motion, such as the Au/Ag/Ni/Ag/Au multi-segment flexible nanomotor oscillates correspondingly at both ends of the flexible Ag and rigid Au due to the oscillation of the magnetic segment (Ni) in the center of the structure under the oscillating magnetic field. (Fig. 8c) [137]. In addition, in the rotating magnetic field, the magnetic material originally rotates along the direction perpendicular to its easy axis of magnetization, and the addition of the flexible material disturbs the balance of rotation, and finally realizes an asymmetric propeller motion, such as Au/Ni/Ag flexible motor (Fig. 8b) [43], Au/PPy/Ni flexible motor [138] and Ni/Ag two-segment flexible motor (Fig. 8d) [24].

## Application of 1D Micro/Nanomotors

As a new micro/nanotool, micro/nanomotor can be flexibly designed with its unique structure and material composition for different application systems, which has been proved to be applicable in many fields [162–165]. In particular, micro/nanomotors can be designed to perform tasks that are difficult to accomplish by conventional means at low cost, such as targeted delivery [144], gene editing [166], and detection [125]. 1D micro/nanomotor has a large aspect ratio and functional area, and the structure and material composition can be flexibly adjusted according to the application needs, which means that 1D motor will be easier to obtain the expected driving mode and functional space. These advantages make 1D motor more popular in many application fields, mainly focusing on biomedicine [146, 167] and environmental applications [99, 168]. In this section, we will mainly show the applications of 1D micro/nanomotors in the fields of cargo transportation, detection, catalytic degradation and microbial treatment.

### Delivery of Cargo

Micro/nanomotors are an emerging carrier at the nanoscale, and cargo transportation is one of its important applications. Generally, stimuli-responsive materials are integrated into micro/nanomotors as a means of cargo delivery and controlled release [169, 170]. According to the type of goods, it can achieve a variety of different purposes, with great flexibility in various fields. Compared with motors of other structures, the advantage of 1D micro/nanomotors in the field of cargo transportation lies in that its aspect ratio is relatively large. Under the same kinetic energy, the contact area between the motor and the obstacle is relatively small, which can generate greater pressure, which is more conducive to crossing various barriers. Therefore, 1D micro/nanomotors are more favored by researchers when crossing obstacles such as cell membranes.

**Fig. 9:**
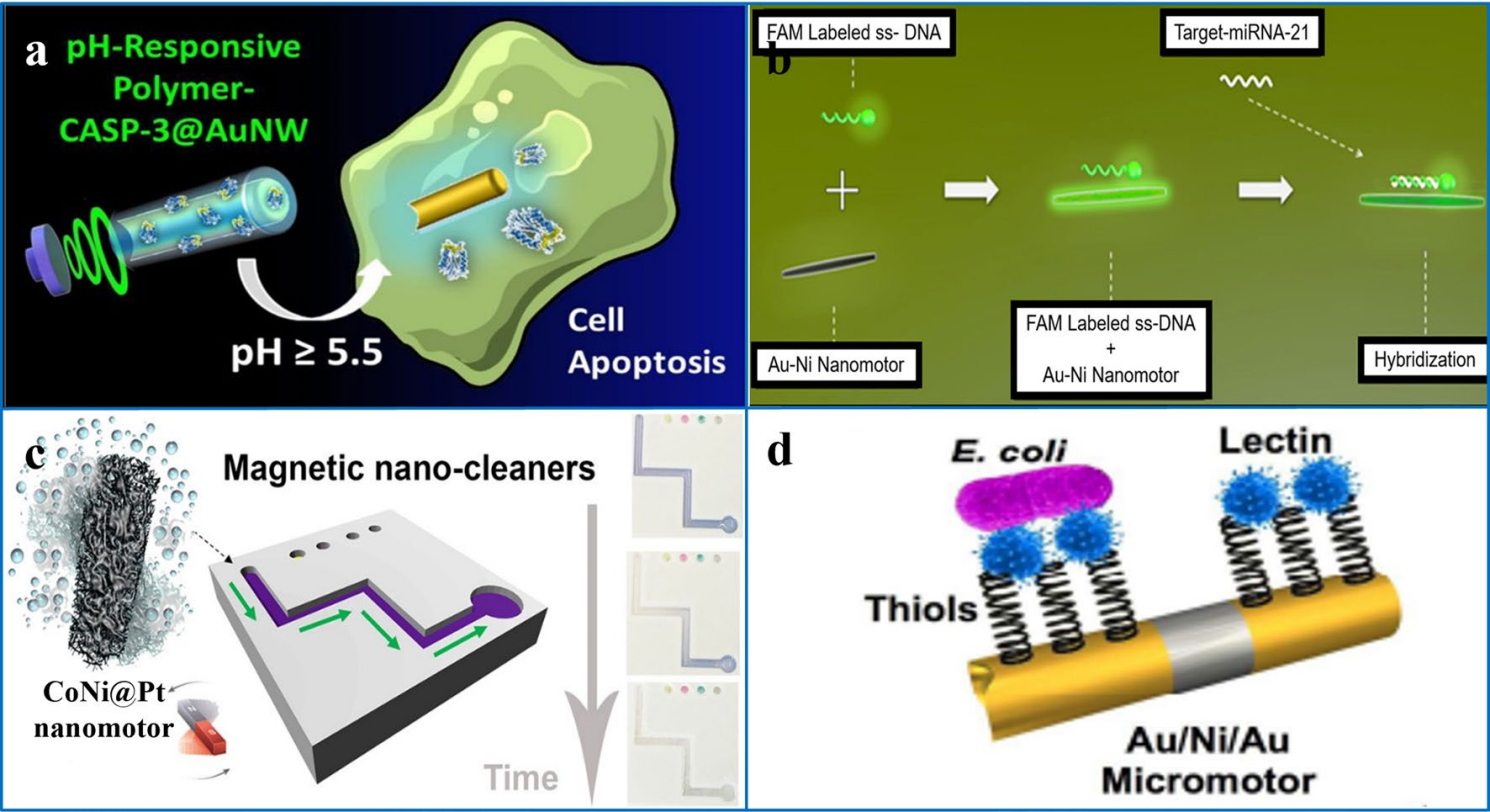
Applications of 1D micro/nanomotors in different fields. **a** Au nanorod motor loaded with CASP-3 into cells and released into cells, leading to rapid cell apoptosis. Reprinted with permission from Ref. [39]. Copyright (2017) American Chemical Society. **b** Detection of cancer biomarker microRNA-21 with Au/Ni nanowires. Reprinted with permission from Ref. [125]. Copyright (2021) American Chemical Society. **c** Magnetic mesoporous CoNi@Pt nanorod degradation contaminants. Reprinted with permission from Ref. [171]. Copyright (2017) American Chemical Society. **d** E. coli captured by Au/Ni/Au micromotor modified concanavalin A. Reproduced with permission from Ref. [146]. Copyright (2017) Royal Society of Chemical

For example, a 1D nanomotor composed of gold nanorods and a pH-responsive polymer coating, loaded with CASP-3, can enter cells and sense intracellular pH, and when the pH is too high, the polymer coating is dissolved, thereby releasing the active CASP-3 enzyme directly into the cytoplasm, resulting in rapid cell apoptosis. (Fig. 9a) [39]. In addition to delivering drugs into cells, researchers have also loaded 1D micro/nanomotors with other cargoes, such as CRISPR-associated protein 9 (Cas9), an attractive genome editing tool called ribonucleic acid (RNA). Gold nanorods loaded with Cas9/sgRNA complexes directly penetrated the surface of B_16_F_10_ cells expressing green fluorescent protein (GFP) by sonication. At the plasma membrane, Cas9/sgRNA is released intracellularly, enabling efficient GFP knockout [144].

### Detection

The development of micro/nanomotors provides an interesting tool for monitoring, analyzing and sensing pollutants and biochemical entities. Combining micro/nanomotors with current detection or analysis methods can speed up processing times and improve analytical results for a variety of analytical scenarios, including environmental pollutants and various biochemical analytes [172–174]. In addition, micro/nanomotor-based analysis can provide real-time and on-demand analysis with higher precision and accuracy to address complex analytical challenges with small sample volumes. Generally speaking, micro/nanomotors used for detection need to modify some substances with special functions, and the high aspect ratio of 1D motors provides an excellent modifiable area, making it a popular choice for various detection experiments.

For example, functionalized gold nanorod nanomotors coated with graphene oxide and modified dye-labeled single-stranded DNA can be used to specifically detect HPV16 E6 mRNA transcripts in cells [145]. As mentioned earlier, in many experiments where motors enter cells, 1D micro/nanomotors are easier to enter living cells than other shapes of micro/nanomotors, because their contact area is smaller, making them more convenient to complete various tasks [39, 126]. For example, dye-labeled single-stranded DNA (ssDNA probe)-modified Au/Ni nanomotors can target an important cancer biomarker microRNA with a linear range of 0.01–25 nM-21 (miRNA-21), for sensitive and selective detection (Fig. 9b) [125].

### Catalytic Degradation

One of the impacts of human activities on nature is environmental pollution, and some even affect human health. Therefore, removing toxic and harmful pollutants from the living environment has also become an appealing application. The emergence of micro/nanomotors provides a new idea to solve this problem. For example, when facing some locations that are difficult to reach by conventional means or dealing with pollutants with low concentrations, micro/nanomotors can show their unique advantages. 1D micro/nanomotors are also popular in this field due to their relatively simple fabrication process and convenient structural design.

Self-propelled micro/nanomotors using either available fuels in the environment or extrinsic fuels is a promising strategy for the catalytic degradation of pollutants. Among them, the most used fuel H_2_O_2_ can also be used as an oxidant to generate highly reactive hydroxyl radicals to degrade organic pollutants. For example, 1D micromotors prepared from radishes can catalyze the decomposition of hydrogen peroxide because they are rich in catalase and peroxidase. While hydrogen peroxide plays a dual role in propulsion and purification, serving as a co-substrate for automotive fuel and phenol conversion, respectively, the design was successfully used to accelerate the removal of phenolic compounds [175]. Another advantage of 1D motors is that it is easier to design new functionalized fragments in the original system. For example, after incorporating magnetic materials, the mesoporous CoNi@Pt nanorods not only exhibited excellent catalytic performance, but also possessed the magnetic guiding function (Fig. 9c) [171]. These catalytic nanorods can act as directionally controllable nano-cleaners to degrade pollutants (4-nitrophenol, rhodamine B, methylene blue, etc.) in the presence of borohydride. Their magnetism can control the velocity and direction of the entire contaminated solution by degrading different contaminants in their path.

### Microbial Treatment

Contamination of drinking water by bacteria or other pathogenic microorganisms is a major public health problem due to rising antimicrobial resistance. In addition, the increasing threat of multidrug-resistant strains to traditional antibiotic therapies poses a significant health risk globally and drives the demand for novel antimicrobial therapies. The development of micro/nanomotors could provide humans with a tool to deal with bacterial outbreaks related to water disinfection and food safety. Many different types of materials, such as silver and its compounds [176], chitosan [177, 178], lysozyme [179], monolayer graphene [72, 180], and photosensitizers [181], can be used as biocides to functionalize micro/nanomotors. 1D motors are favored by researchers because of their large functional area and convenient structural design. For example, (PPy)–COOH/PEDOT/Ni/Pt micromotors modified with anthrax spore (anti-Bacillus) antibodies were developed to selectively capture and destroy anthrax spores. Antibody-modified micromotors were able to target and capture anthrax-mimicking spores while moving in the presence of excess non-target *Staphylococcus* *aureus* and *Escherichia coli* in PBS and water [182].

In addition to killing pathogenic bacteria, functionalized micro/nanomotors provide a great tool for real-time microbial manipulation, which may lead to the development of advanced analytical platforms for water quality testing [183, 184], food safety [185], and more. For example, acoustically driven, magnetically guided Au/Ni/Au 1D micromotors were used to target *E. coli* with target vectors [146]. After functionalization of the lectin receptor Concanavalin A, *E. coli* was captured with 96% efficiency, loading an average of 3–4 targets (3–4 targets per motor). Simple separation of the target and micromotor is also possible (Fig. 9d).

## Summary and Outlook

With the advantages of relatively simple preparation process, flexible design, and large surface area, 1D micro/nanomotors are favored by researchers and are widely used in biomedical, environmental management, and sensing fields. This review systematically summarizes the reported 1Dmicro/nanomotors and introduces the conventional preparation methods of these 1D micro/nanomotors, including template assisted electrodeposition, vapor deposition, winding, hydrothermal synthesis and other methods. Then, 1D micro/nanomotors are classified according to their structures, and the driving mechanisms of each kind of 1D micro/nanomotors are introduced. Finally, practical applications of 1D micro/nanomotors in cargo transportation, detection, catalytic degradation, microbial treatment, etc*.* are summarized.

As a new research field, 1D micro/nanomotors have shown unique advantages in many aspects, but they still face many challenges: (1) Higher energy conversion efficiency. More powerful propulsion and higher movement speed, for the motor, means a stronger ability to break through obstacles and a more efficient ability to perform tasks. At present, the fastest artificial micro/nanomotor can reach 11 mm/s, which is also a 1D motor [186]. But this data was tested in a water environment. In the face of complex real-world applications, such as blood in blood vessels, it is necessary to overcome not only the viscosity of the blood, but also the flow rate of the blood. Therefore, the development of micro/nanomotors with higher energy conversion efficiency and stronger power is still ongoing, which is an important challenge. (2) Better direction control. Motion direction control is another important basis for the application of motors. As mentioned above, currently, there are only two ways to control the direction of 1D motor: magnetron control [187] and light control [117]. Therefore, in the face of complex real application scenarios, the current directional control methods are relatively limited. On the other hand, these two control methods are limited by the current motor structure and materials, and cannot achieve very accurate responsive control. Therefore, more and better control methods need to be developed. (3) More sophisticated preparation methods. The most unique advantage of micro/nanomotors is that they are extremely small and movable. Therefore, smaller micro/nanomotors have larger specific surface area and wider active area, which means better application value. Especially in the biomedical field, the size of the motor is critical to its application. However, the current smallest 1D motor can only achieve a diameter of 20 nm and a length of 150 nm [86]. In addition, there is only one way to achieve it. Therefore, a more refined preparation process needs to be developed urgently.

Therefore, we believe that 1D micro/nanomotor can be improved in the following aspects: (1) Develop new materials. At present, 1D micro/nanomotors are mainly prepared based on metals and metal oxides or polymers. Despite their respective advantages, in order to develop more powerful and widely used 1D motors, new materials for building motors need to be further explored. We can consider this issue from 3 perspectives. On the one hand, exploring new synthetic materials, such as supramolecular materials, porous materials, carbon materials, liquid crystal materials, etc., which can be designed to prepare corresponding functional materials for specific needs or applications. On the other hand, looking for natural materials. Find new ideas from the composition materials of animals and plants in nature, and make full use of the characteristics of natural materials endowed by nature, in order to improve the motor performance or to expand the application prospects of motors. Finally, developing composite materials. In this way, it is possible to achieve both the needs of the specific performance of artificial materials and the biological advantages of the natural material to the motor. (2) Emphasis on sub-microstructure. As mentioned above, the 1D micro/nanomotor has little resistance due to its inherent structural advantages. Therefore, the fastest micro/nanomotors at present belong to the 1D motor. However, the motion performance of the motor is closely related to the structure of the motor. To further improve the energy conversion efficiency and improve the power of the motor, we must not only consider the overall 1D structure of the motor, but also consider the sub-microscopic structure of the motor, such as the Janus nanostructure. The interface structure of the two materials of the rod nanomotor, the surface morphology of the inner wall of the micro-tubular motor, etc. Based on the mastery of the material properties, it is beneficial to further improve the motor performance through a more refined structural design. In addition, learning from biological structures in nature and using biomimetic structures to achieve efficient energy conversion, is also a very important strategy needed to be considered. (3) Combine nanofabrication technology. According to the preparation methods introduced above, it can be seen that many advanced micro-nano processing technologies have been applied to the preparation of 1D micro/nanomotors. In order to solve the current predicament. On the one hand, it is necessary to rely on the emergence of new nanotechnology to apply to preparation of 1D nanomotors. On the other hand, more importantly, we can try to make the existing technology effective fusion. Reasonably joint use current nanotechnologies will have unexpected advantages. For example, the smallest 1D motor at present broke through the preparation limit of conventional nanofabrication technology through the effective combination of template method and ALD [86], and brought such a shocking product. How to use existing nanotechnology to fabricate finer micro/nanomotors, this work provides us with good inspiration.

The most promising prospect of 1D micro/nanomotor depends on its easily adjustable structure and excellent performance when crossing the barrier. The intelligent 1D micro/nanomotors in the future should have a more sophisticated structure and more diverse functions, as well as a perfect control and monitoring system, and have more safe and efficient practical applications in biomedical and environmental treatment fields. We hope that our review can provide guidance for the design of the next generation of intelligent 1D micro/nanomotors to achieve wider practical applications and unprecedented performance. In the near future, we believe that 1D micro/nanomotors can become cost-effective and powerful micro/nanotools, ushering in the dawn of the nanotechnology revolution.
